# Still facial photographs of long-term meditators are perceived by naïve observers as less neurotic, more conscientious and more mindful than non-meditating controls

**DOI:** 10.1371/journal.pone.0221782

**Published:** 2019-08-28

**Authors:** Simon B. Goldberg, Matthew Hirshberg, Lawrence Y. Tello, Helen Y. Weng, Lisa Flook, Richard J. Davidson

**Affiliations:** 1 Department of Counseling Psychology, University of Wisconsin–Madison, Madison, WI, United States of America; 2 Center for Healthy Minds, University of Wisconsin–Madison, Madison, WI, United States of America; 3 National Eye Institute, National Institutes of Health, Bethesda, MD, United States of America; 4 Osher Center for Integrative Medicine, University of California, San Francisco, San Francisco, CA, United States of America; 5 Learning Policy Institute, Palo Alto, CA, United States of America; 6 Department of Psychology, University of Wisconsin–Madison, Madison, WI, United States of America; 7 Department of Psychiatry, University of Wisconsin–Madison, Madison, WI, United States of America; Virginia Commonwealth University, UNITED STATES

## Abstract

The impact of meditation training on self-report psychological variables is well-established. Although meditation training is purported to have interpersonal impacts, whether naïve observers perceive differences associated with long- and short-term meditation training is largely unknown. The current study provided a stringent test of this possibility through observer ratings of a very thin slice of expressive behavior: still photographs. Photographs were drawn from a larger study investigating differences between long-term meditators (LTM) and meditation naïve participants (MNP) who were exposed to one of three experimental conditions. Photographs of ninety-nine targets (16 LTMs, 83 MNPs) were taken at baseline, prior to the randomization of MNPs to an eight-week mindfulness meditation course (mindfulness-based stress reduction; *n* = 27), an active control comparison condition (health enhancement program; *n* = 29), or a waitlist control group (*n =* 27) and again after the training period. Pre- and post-intervention photographs were then rated by 25 meditation teachers and 86 undergraduate raters on five domains theoretically linked to meditation training. Results indicated that relative to MNPs, LTMs were rated as less neurotic and more conscientious, mindful, and “comfortable in their own skin” at baseline (*d*s = 0.61 to 0.70, *p*s < .050), although not more agreeable or attractive. Results were largely unchanged when controlling for five observable confounds (age, gender, race/ethnicity, body mass index, attractiveness). No evidence was found supporting experimental effects of short-term meditation training on observer ratings. Thus, it seems that if meditation is associated with observable differences in facial behavior, effects may be limited to long-term training.

## Introduction

There has been a dramatic increase in interest in meditation over the past several decades. Recent data from the National Health Interview Survey showed a three-fold increase in past year meditation use between 2012 and 2017 in the United States (4.1% to 14.3%).[1] Scientific interest in the topic has also increased exponentially. Experimental data from randomized controlled trials (RCTs) have shown reductions in psychiatric symptoms and improvements in well-being in the context of relatively brief (e.g., eight-week) meditation interventions, in both clinical[2,3] and non-clinical populations.[4] A related body of research has examined differences between individuals with extensive meditation experience (i.e., long-term meditators [LTM]) and meditation naïve participants (MNP). Relative to MNPs, LTMs have often shown lower psychiatric symptoms and higher well-being[5] along with a host of biological differences indicating less reactive stress physiology and improved emotion and attention regulation,[6–9] although not always.[10] Meditation training has been linked via meta-analysis to lower neuroticism and higher attention and empathy.[11] Further, dispositional mindfulness, a psychological construct purported to be cultivated in the context of various forms of meditation training (e.g., mindfulness meditation),[12,13] is correlated with several aspects of personality including neuroticism (*r* = -.45), conscientiousness (*r* = .32), and agreeableness (*r* = .22).[14]

Theories on the effects of meditation practice claim that they will be embodied and impact both psychological and physiological variables.[15] Although the effects of meditation practice impacting psychological variables as reported by the practitioner have been well-established,[2,5] little research has examined how meditation practice may impact interpersonal perceptions by others, including close relationships and strangers. Theories suggest that meditation practice enhances a sense of interconnectedness, and may also impact others through greater interpersonal connection and prosocial behavior.[16–20] Most research has been conducted on first-person reports of effects, and rarely report on second-person observer effects, to examine whether the effects of meditation practice can be detectible by others.[21] However, interpersonal perception through second-person observers is important, both as a potential indicator of internal states (e.g., mood, well-being) as well as a proxy for how an individual may be perceived in daily life. Moreover, being perceived more favorably by others (e.g., as happier) may lead to more positive expectations of social interactions,[22] which could in turn produce beneficial inter- and intrapersonal effects. Thus, the domain of interpersonal perception may be one *in* which and *through* which beneficial effects of meditation practice appear. We tested the possibility that meditation training may be associated with differences in second-person observer effects with a stringent criterion, using a thin slice of behavior from still photographs and observers with no familiarity with the participants.

A wide variety of observer rating paradigms have been employed in social psychology. In their review of 263 independent samples, Connelly and Ones[23] demonstrated that observer ratings show predictive validity (e.g., of academic and job performance), incremental to and at times better than self-ratings. Although, the accuracy of observer ratings (i.e., inter-rater agreement, self-other agreement) varied considerably depending on a variety of factors including who was providing the rating (e.g., family member vs. stranger) and the trait being rated (e.g., extraversion vs. neuroticism).

One widely used observer rating paradigm involves ratings made by strangers (i.e., at zero acquaintance), often through brief excerpts or “thin slices” of expressive behavior.[24] Ratings made by individuals who have more information about targets (e.g., family members, coworkers) tend to outperform ratings at zero acquaintance (e.g., when predicting self-ratings of personality[23]). However, zero-acquaintance ratings have high ecological validity: in theory, they reflect how an individual is perceived by strangers, a key aspect of interpersonal perception in daily life.[25,26] Further, from the perspective of evolutionary psychology, the ability to detect personality traits at zero acquaintance has clear adaptive utility (e.g., detection of emotional instability).[27] Within zero-acquaintance ratings, a variety of factors appear to influence the accuracy of ratings, including the type of information available (e.g., audio plus visual vs. text/electronic communication) and the trait being rated (e.g., extraversion vs. neuroticism).[23] The Realistic Accuracy Model (RAM) provides a framework for understanding this variability, noting that accurate judgments of personality require relevant information to be available and both detected and utilized by raters in making judgments.[26,28] This model helps understand why personality traits with higher visibility (e.g., extraversion) are more accurately rated at zero acquaintance than less visible traits (e.g., neuroticism), as well as why accuracy generally increases with the quantity and quality of available information (e.g., audio plus visual information yields more reliable ratings than text/electronic communication).[23] Similarly, the Self-Other Knowledge Asymmetry (SOKA) model highlights how the accuracy of ratings varies across domains, with self-ratings showing higher accuracy than observer ratings for traits low in visibility (e.g., neuroticism).[29]

It is important to note that while observer rating paradigms involving thin slices of behavior at zero acquaintance are intriguing and potentially informative, they should not be interpreted as necessarily measuring reality. Such ratings show only modest correlations with self-ratings (especially for less observable traits like neuroticism, *r* = .08)[23] and can be heavily influenced by stereotypes and other perceiver effects (i.e., rating biases within the individual making the rating).[30] Further, these judgments are not necessarily stable over time, tending to become more accurate as acquaintanceship increases.[31] Nonetheless, ratings at zero acquaintance do provide information about how an individual is perceived by strangers. The incremental validity of such ratings over and above more conventional self-report measures is an issue that still warrants further study.

To our knowledge, only one prior study examined the effect of meditation training on thin slices of behavior using ratings at zero acquaintance. In two small independent samples (*n*s = 26 and 20), Choi, Karremans, and Barendregt[32] found that novice meditators were rated as looking happier after a meditation retreat and experienced meditators were rated as looking happier relative to control participants based on brief video snippets. While intriguing, this work included only one rating dimension, a modest sample size of targets (i.e., those being rated), and a lack of random assignment, limiting conclusions that can be drawn about the experimental effects of meditation training. Further, it is unclear whether an even thinner slice of behavior (e.g., a still photograph, which has been used in other thin slices research),[22] may allow detection of training-related correlates.

The current study sought to address these limitations and explore the boundaries of information required to detect meditation-related differences through second-person observation. We employed one of the thinnest slices of expressive behavior–a still photograph–which provided a more stringent test of the lower limits of information for detecting correlates of meditation training. Relative to videos, photographs are less unlikely to provide potentially confounding contextual cues as to whether an individual engages in meditation practice (e.g., based on the language they use or the topics they discuss). Photographs were obtained in the context of a larger study examining differences between LTMs and MNPs as well as the experimental effects of meditation training through an RCT. Observers included undergraduate raters as well as meditation teachers to represent a range of perspectives from naïve to expert. Assessments were made across several dimensions theoretically linked to meditation practice and embodiment,[33] including three of the Big Five personality traits.[14] Of note, rating dimensions were selected based on their theoretical relevance to meditation training and included several constructs reflecting internal states despite their low visibility (e.g., neuroticism).

## Materials and methods

### Participants

This study was approved by the University of Wisconsin-Madison Institutional Review Board. Written consent was obtained from participants.

#### Targets

Targets were recruited as part of a larger study investigating the effects of long- and short-term meditation training.[9,34] Participants included as targets in the current study consented to have their photographs used in future research. A sample of 16 long-term meditators (LTM; age = 50.62, *SD* = 9.56 years, 8 female, 14 non-Hispanic white, 10 attended graduate school, see Table 1 for full demographics) were recruited at meditation centers and through mailing lists. To be included, LTMs had to have practiced Vipassana and compassion/loving-kindness meditation for at least three years, have a daily practice of 30 minutes or more, and have attended at least three residential meditation retreats lasting five days or more (see Rosenkranz et al., 2016). LTMs had an average of 8,774 lifetime hours of meditation practice (range = 1,439 to 32,612; *SD* = 7,041). A sample of 83 meditation naïve participants (MNP; age = 48.79, *SD* = 11.13 years, 53 female, 76 non-Hispanic white, 42 attended graduate school) matched on age and gender were recruited in the Madison, WI area using online and print media. Demographic matching within the larger trial was focused on age and gender due to the association between these variables with a variety of neurobiological and psychological variables. Only MNPs who provided images at both pre- and post-test were rated. The LTM and MNP groups did not differ by age, gender, race/ethnicity, or education (*p*s>.050). Participants in both groups were excluded if they had used psychotropic medications, had a psychiatric diagnosis in the past year, or had a history of bipolar or schizophrenic disorders, brain damage, or seizures.

**Table 1:** Sample demographics.

	Targets		Raters	
Demographic Variable	LTM (n = 16)	MNP (n = 83)	Undergraduates (n = 86)	Meditation Teachers (n = 25)
**Age, mean(SD)**	50.62 (9.56)	48.79 (11.13)	19.07 (3.37)	52.80 (12.25)
**Female, n(%)**	8 (50)	53 (63.9)	54 (62.8)	9 (36.0)
**Race/ethnicity**				
non-Hispanic white, n(%)	14 (87.5)	76 (91.6)	55 (64.0)	20 (80.0)
non-Hispanic black, n(%)	0 (0.0)	0 (0.0)	5 (5.8)	1 (4.0)
Asian, n(%)	2 (12.5)	2 (2.4)	15 (17.4)	0 (0.0)
Native American, n(%)	0 (0.0)	0 (0.0)	1 (1.2)	0 (0.0)
Hispanic, any race, n(%)	0 (0.0)	4 (4.8)	1 (1.2)	1 (4.0)
More than one race/ethnicity, n(%)	0 (0.0)	1 (1.2)	8 (9.3)	1 (4.0)
Did not want to respond, n(%)	0 (0.0)	0 (0.0)	1 (1.2)	2 (8.0)

LTM = long-term meditator; MNP = meditation naïve participant.

#### Raters

Two samples of raters were recruited. A sample of 25 Buddhist meditation teachers (age = 52.80, *SD* = 12.25 years, 9 female, 20 non-Hispanic white) were recruited through meditation centers. Inclusion criteria were self-identification as a meditation teacher and experience leading residential meditation retreats. A sample of 86 undergraduate raters (age = 19.07, *SD* = 3.37 years, 54 female, 55 non-Hispanic white) were recruited through psychology courses at the University of Wisconsin–Madison.

### Procedure

#### Intervention

Following baseline assessment, which included collection of photographs of spontaneous emotion (see below), MNPs were randomly assigned to one of three conditions: mindfulness-based stress reduction (MBSR),[15] health enhancement program (HEP),[34] or a waitlist (WL) control condition. MBSR is a standardized, eight-week mindfulness intervention involving instruction in formal (e.g., sitting meditation) and informal (e.g., attentiveness during daily life) mindfulness practice. MBSR was delivered in the typical group format by experienced MBSR instructors. HEP is an active control condition designed specifically to match MBSR as closely as possible, while not including mindfulness content. HEP includes mild physical activity, functional movement, nutrition education, and music and imagery designed to enhance psychological health.[34] HEP was delivered in an eight-week, group format by instructors with expertise in HEP content but no background in mindfulness. Participants in the WL condition received no intervention. The current MNPs were a subset of a sample of 130 participants enrolled in the larger trial.[34] Among the 86 MNP who provided consent to have their photographs used in future research, 27 were assigned to MBSR, 29 to HEP, and 27 to WL.

#### Photographs

Prior to randomization, LTMs and MNPs were photographed by a research assistant blind to study condition (i.e., LTM vs. MNP). MNPs were photographed again post-intervention. Photographs were taken as part of study visits that were equivalent for LTMs and MNPs. Participants were told “we’re just going to take your photo,” allowing a spontaneous facial expression (i.e., not restricted to a neutral expression). Spontaneous facial expressions have been shown to yield more accurate observer ratings of personality.[35] Color photographs were subsequently cropped at the neck and resized so that participants’ heads were approximately the same size.

#### Rating paradigm

Raters, blind to study condition, provided ratings of still photographs. Both undergraduate and meditation teacher raters completed ratings through online surveys. Undergraduate raters completed surveys in the laboratory and meditation teachers completed surveys remotely. Each item was phrased as follows, with the respective trait varied across items: “How [trait] is this person? Please respond from 1 (not at all) to 7 (very).” We chose traits to be rated due to their relationship to well-being and embodiment, both of which are theoretically cultivated through meditation practice.[4,14,33] Of note, these dimensions were selected based on their potential association with meditation training, rather than their visibility (e.g., extraversion was not rated). Six items were drawn from the Ten Item Personality Inventory[36] assessing the Big-Five personality dimensions of conscientiousness, agreeableness, and neuroticism (two items for each). Two novel items assessed mindfulness (“mindful”) and embodiment (“comfortable in their own skin”). One item assessed attractiveness as a potential confound.[22]

Due to the large number of photographs and dimensions being assessed, six separate surveys were created (see Table 2). Across four separate samples, undergraduate raters rated two items for each of the three Big Five personality traits (conscientiousness, agreeableness, neuroticism) as well as attractiveness and “comfortable in their own skin.” We anticipated greater difficulty recruiting meditation teachers, so planned to have them rate a subset of items in order to increase reliability of available ratings. Across two separate samples, meditation teacher raters rated a single item for neuroticism and agreeableness, along with “comfortable in their own skin” and “mindful.”

**Table 2:** Descriptions of six surveys.

Survey	Sample	Traits assessed
1	21 undergraduates	^a^Calm/emotionally stable
2	21 undergraduates	Attractive
3	22 undergraduates	^a^Anxious/easily upset, ^a^Sympathetic/warm, ^a^Dependable/self-disciplined
4	22 undergraduates	^a^Critical/quarrelsome, ^a^Disorganized/careless, Comfortable in their own skin
5	14 meditation teachers	^a^Anxious/easily upset
6	11 meditation teachers	^a^Sympathetic/warm, Comfortable in their own skin, Mindful

^a^Six items drawn from Ten Item Personality Inventory (Gosling et al., 2003) assessing conscientiousness, agreeableness, and neuroticism.

### Data analysis

Inter-rater reliability of observer ratings was determined using Shrout and Fleiss’s[37] intra-class correlation coefficient (ICC) for fixed judges (i.e., ICC3, in which each target is rated by all judges; see S1 Table for sample R code). Observer ratings were then aggregated across raters and within target, yielding a single rating per target for each item. For items assessing personality dimensions, a composite score was computed by averaging across the two items (reverse scored as appropriate). To compare LTMs and MNPs at baseline, regression models were constructed predicting observer ratings from LTM status. Subsequent models controlled for observable confounds (age, gender, race/ethnicity, observer-rated attractiveness, body mass index). Analysis of variance (ANOVA) models examined intervention-related changes in observer ratings across the three randomized conditions (MBSR, HEP, WL). Our study was powered to detect large to moderate differences between LTM and MNP groups and large differences between the three randomized conditions.[38] However, *a priori* power calculations were for the larger trial from which these data are drawn. Specifically, the original study was powered to detect large to moderate (*d* = 0.74) between group effects based on prior fMRI studies. The larger trial proposed samples of 36 participants per group (total *n* = 144 across four groups) to allow for attrition. To control for potential Type I error due to conducting tests across six target dimensions in our primary analyses (i.e., LTM vs. MNP at baseline, intervention-related time X group effect for MNPs), we controlled for false discovery rate using Benjamini and Hochberg’s[39] method implemented using the ‘p.adjust’ function in R.[40] This method provides a more powerful alternative to Bonferroni-type adjustments and has been recommended for theory-driven contexts specifically.[41]

The larger trial from which these data were drawn was not pre-registered. At the time the larger trial was funded (2008), pre-registration was not widespread. A variety of self-report, behavioral, and neuroimaging data were collected as part of the larger trial and are not reported here. Similarly, the data collected via observer ratings reported here were not pre-registered. However, between-group comparisons (i.e., LTMs vs. MNPs, MBSR vs. HEP vs. waitlist) on personality dimensions and embodiment items were planned *a priori*. Along with rating attractiveness, raters also assessed “old” and “healthy” as two additional potential confounds, but those data are not reported here. An additional rating task was completed by a subsample of raters in which observers were shown a pre- and post-test photograph and were asked to choose which occurred following a well-being intervention. There was no indication that observers were able to predict post-intervention photographs above chance (overall, or for the active groups [MBSR, HEP] specifically). These data are not reported here.

## Results

All items showed adequate inter-rater reliability (ICC≥.75).[42] The two item personality scales showed adequate internal consistency reliability (α≥.80). Ratings from meditation teachers and undergraduates were highly correlated (*r*s≥.66, *p*s < .001) and rater type (i.e., meditation teacher vs. undergraduate) did not moderate the association between group (LTM vs. MNP at baseline, MBSR vs. HEP vs. WL in ANOVAs) and ratings (*p*s>.050). Ratings were therefore combined to increase reliability.[37]

**Fig 1:**
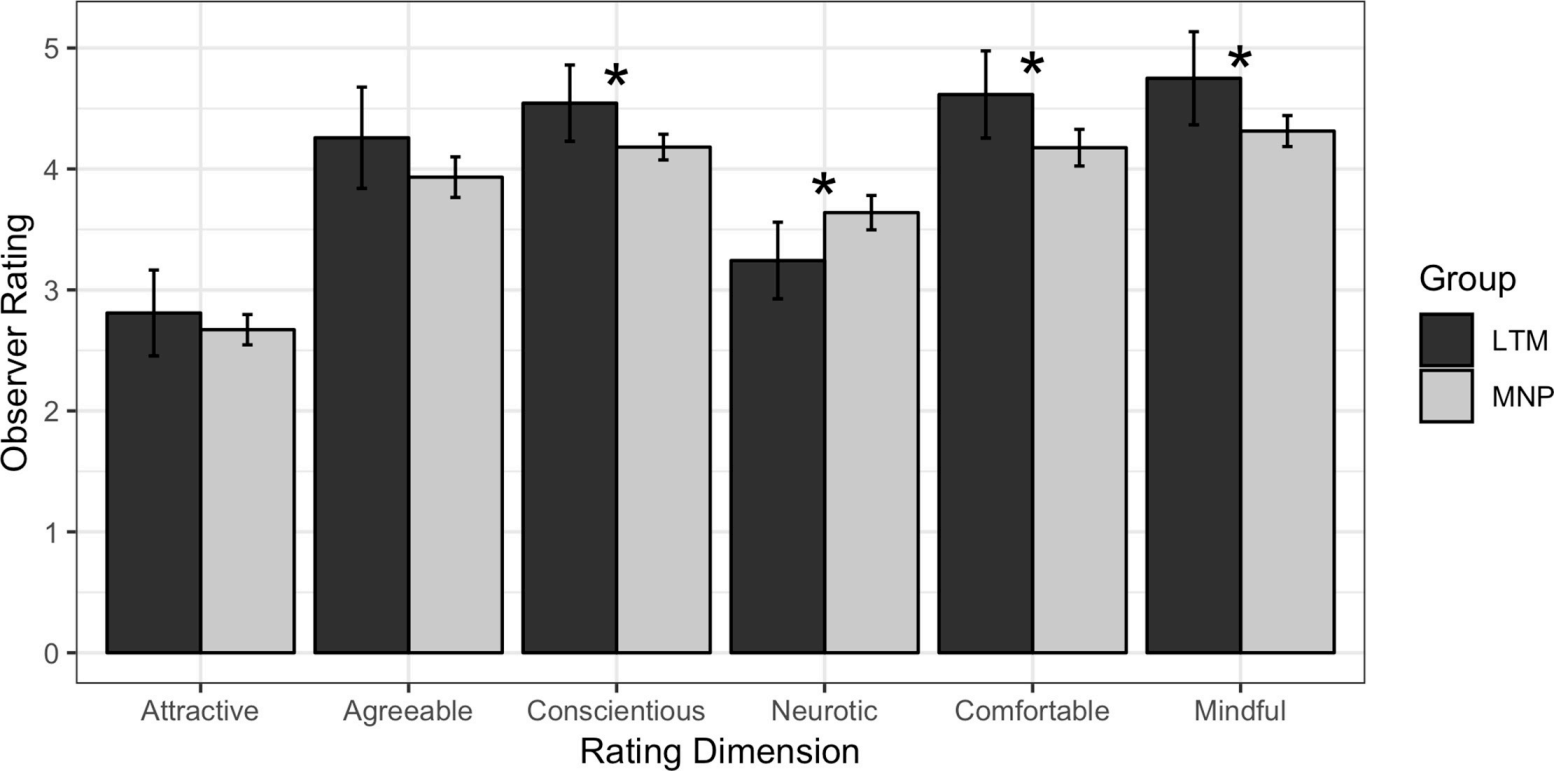
Long-term meditation (LTM) practitioners are rated as less neurotic and more conscientious, “comfortable in their own skin,” and mindful compared to meditation naïve participants (MNP). Target sample includes 16 LTMs and 83 MNPs. Rater sample includes 25 meditation teachers and 86 undergraduates.

Means and standard deviations for the six constructs assessed are included in Tables 3 and 4. At baseline, LTMs were rated more highly than MNPs on conscientiousness, comfortable in their own skin, and mindful and were rated lower than MNPs on neuroticism (absolute value of *d*s = 0.61 to 0.70, *p*s < .050; Fig 1, Table 3). No differences were observed for ratings of agreeableness or attractiveness (*p*s>.050). Results were unchanged controlling for observable confounds, with the exception of two models (LTM status predicting comfortable and agreeableness) in which LTM status became a marginally significant predictor (*p*s = .085, .089, respectively) when confounds were statistically controlled. A sensitivity analysis separated ratings from undergraduates and meditation teachers. Using undergraduate ratings only, LTMs were rated as more conscientious and comfortable than MNPs, but no longer less neurotic (*p* = .052). Using meditation teacher ratings only, LTMs were rated as less anxious and more mindful, but no longer more comfortable (*p* = .161).

**Table 3:** LTMs versus MNPs at baseline.

Rating Domain	LTM	MNP	*d*	*p*
Attractive	2.81 (0.72)	2.67 (0.58)	0.23	0.406
Agreeable	4.26 (0.85)	3.93 (0.78)	0.42	0.162
Conscientious	4.54 (0.64)	4.18 (0.49)	0.70*	0.039
Neurotic	3.24 (0.65)	3.64 (0.66)	-0.61*	0.045
Comfortable	4.62 (0.74)	4.18 (0.70)	0.62*	0.045
Mindful	4.75 (0.79)	4.31 (0.60)	0.69*	0.039

Sample sizes: LTM = 16, MNP = 83. LTM = Long-term meditation practitioners; MNP = meditation naïve participants at pre-test; *d* = Cohen’s *d*; Comfortable = “comfortable in their own skin”; Big-Five personality dimensions assessed using two-item scales drawn from the Ten Item Personality Inventory;[36] *p* = *p*-values from independent samples t-test, adjusted for false discovery rate;[39]

*p < .050.

**Table 4:** Pre-post comparison for MNPs randomized to three conditions.

	MBSR			HEP			Waitlist			
Rating Domain	Pre	Post	*d*	Pre	Post	*d*	Pre	Post	*d*	*p*
Attractive	2.66 (0.64)	2.62 (0.65)	-0.06	2.56 (0.52)	2.55 (0.54)	-0.02	2.81 (0.58)	2.77 (0.57)	-0.07	0.991
Agreeable	3.66 (0.72)	3.69 (0.74)	0.04	3.93 (0.81)	4.17 (0.79)	0.3	4.20 (0.74)	4.25 (0.82)	0.06	0.978
Conscientious	4.07 (0.40)	4.08 (0.50)	0.02	4.08 (0.58)	4.18 (0.57)	0.17	4.39 (0.42)	4.36 (0.54)	-0.06	0.978
Neurotic	3.87 (0.64)	3.85 (0.61)	-0.03	3.65 (0.69)	3.39 (0.62)	-0.4	3.40 (0.58)	3.36 (0.62)	-0.07	0.978
Comfortable	3.94 (0.71)	4.01 (0.71)	0.1	4.18 (0.71)	4.41 (0.70)	0.33	4.41 (0.64)	4.51 (0.72)	0.15	0.978
Mindful	4.33 (0.69)	4.28 (0.57)	-0.08	4.30 (0.56)	4.40 (0.61)	0.17	4.31 (0.55)	4.30 (0.60)	-0.02	0.978

Sample sizes: MBSR = 27, HEP = 29, waitlist = 27. MNP = meditation naïve participants; MBSR = Mindfulness-Based Stress Reduction; HEP = Health Enhancement Program; Pre = pre-test; Post = post-test; *d* = within-group (i.e., pre-post) Cohen’s *d*; Comfortable = “comfortable in their own skin”; Big-Five personality dimensions assessed using two-item scales drawn from the Ten Item Personality Inventory;[36] *p* = *p*-values from time X group ANOVA models, adjusted for false discovery rate;[39] *p < .050.

Time by group interaction terms were used to model changes in observer ratings for MNPs over the course of intervention (MBSR, HEP, WL; Table 4). No time by group effects were observed on any of the six dimensions assessed (*p*s>.050). Results remained unchanged controlling for observable confounds. Exploratory *post hoc* analyses examined time by group interactions for each pairing (i.e., MBSR vs. HEP, MBSR vs. WL, etc.) and pre-post within-group changes. No time by group effects or pre-post within-group changes were observed (*p*s>.050).

## Discussion

The current study examined the association of long- and short-term meditation training with observer ratings of personality and related dimensions. Photographs were taken of LTMs and MNPs at baseline, and again following MNPs’ completion of training based on randomization to a mindfulness meditation training (MBSR), an active control condition (HEP), or a waitlist. High inter-rater reliability was obtained across all rating dimensions. Results supported the possibility that long-term meditation training may be linked with perceived differences in neuroticism and conscientiousness by strangers despite minimal information. Significant differences in the moderate range were observed on ratings of these personality traits, as well as on novel items intended to assess constructs specific to meditation (comfortable in their own skin, mindful). While results do not imply these reflect actual differences between LTMs and MNPs on these traits (and the broader interpersonal perception literature suggests ratings of internal states by strangers may not reflect actual differences),[23,27] they also do not appear to reflect merely globally positive ratings of LTMs. In particular, LTMs and MNPs did not differ on ratings of agreeableness and attractiveness. Further, results did not appear to be primarily driven by differences in observable confounds (e.g., age, attractiveness). Results were most robust when ratings from undergraduates and meditation teachers were combined.

In contrast, no evidence was found suggesting effects of short-term meditation training on observer ratings. In fact, neither MBSR nor the active control condition (HEP) differed from the waitlist group in observer-rated changes over the course of the eight-week interventions. Thus, if meditation is associated with changes in observers’ perception of personality (which our design cannot demonstrate for LTMs due to non-random assignment), these effects may be restricted to long- rather than short-term training.

The possibility that long-term meditation practice may be associated with observer’s perception of neuroticism and conscientiousness is intriguing, albeit qualified by a lack of randomization of participants to the LTM and MNP conditions. Interpersonal perceptions matter; they show incremental validity beyond self-report for predicting a variety of outcomes (e.g., teacher effectiveness, academic performance)[23,24] and are not confounded with known biases in self-report (e.g., social desirability) that may be particularly pernicious when assessing internal processes impacted by meditation training (e.g., mindfulness).[13,43] Our results support the notion that long-term meditation practice is associated with more favorable perceptions by others in still photographs, on dimensions with potentially important intra- and interpersonal consequences.[44] The ways in which these perceptions made from still photographs may relate to perception in daily life is not clear from the current study, of course. Nonetheless, it is possible that LTMs may experience a more welcoming interpersonal environment, vis-à-vis others’ initial impressions.[22] Should that be the case, improved interpersonal interactions could be both an outcome of long-term practice as well as a mechanism through which long-term practice yields benefits in other domains (e.g., well-being, quality of life).

It is important to highlight that perceived differences between LTMs and MNPs were evident using a very small sample of expressive behavior (i.e., still photographs). While this may reflect the potency of potential differences (i.e., they could be detected from minimal information), the observed effect should be contextualized within the broader thin slices and zero acquaintance ratings literature. Meta-analytic evidence has highlighted the modest reliability for ratings by strangers of internal states based on still visual cues (e.g., *r*_*rr*_ = .25 for emotional stability) as well as the low self-other agreement for ratings by strangers of internal states (e.g., *r* = .08 for emotional stability, *r* = .12 for openness to experience).[23] Therefore, perceived differences must be interpreted as simply that–differences in perception–and may or may not reflect differences in LTMs’ and MNPs’ actual internal states. This is particularly so given the small amount of information available to observers (e.g., still photographs). Again, still photographs are clearly not analogous to how interpersonal perception typically occurs within daily life–we almost always have access to considerably more information when interacting with others. However, our results nonetheless suggest that observers somehow perceived LTMs more favorably across several domains highly relevant to meditation training, despite the limited information provided.

It would be worthwhile continuing to unpack these initial findings in future studies. One potentially fruitful future direction implied by the Realistic Accuracy Model would be obtaining samples of behavior in situations with higher relevance to the traits being assessed which may be more likely to provide available relevant information that could be detected and utilized by raters to make valid judgments.[26] Recent work has shown that ratings of neuroticism are more accurate in trait-relevant situations (e.g., socially stressful situations).[27] A potential future direction could be obtaining samples of behavior in such situations (e.g., Trier Social Stress Task),[9,45] ideally within the context of random assignment to short-term meditation training. Perhaps tasks could be used and/or developed that provide information about other aspects impacted by meditation training assessed here (e.g., mindfulness, embodiment, conscientiousness). It could also be illuminating to determine which aspects of behavior raters are using as cues to differentiate LTMs and MNPs or the short-term effects of training. This could be done using machine learning and social sensing technologies.[46] Future studies could also continue to explore potential signals detectable through still photographs, perhaps opening the door to measurement strategies that have not been widely implemented but could hold promise for the detection of emotional signals in daily life (e.g., examining facial expressions obtained through smartphones).

A future study with a larger sample of LTMs could also explore potential dose effects of meditation on observer ratings of personality across LTMs (i.e., is more training associated with more positive perceptions). The small sample of LTMs in the current study (*n* = 16) prohibited a proper assessment of this possibility.

The lack of short-term training effects is harder to interpret and could be due to a genuine lack of impact or an insensitivity of our observer-rated measures to these potentially more subtle changes. Pre-post analyses also relied on a subset of participants, which reduced our statistical power to detect effects (although very small pre-post effects suggest power is not the sole issue). It seems the most prudent conclusion is that short-term meditation training does not impact interpersonal perceptions of personality observable through photographs. As noted above, it could be valuable to assess the impact of short-term training in future studies through samples of behavior drawn from contexts more likely to provide cues reflecting target constructs of interest (e.g., socially stressful situations, tasks requiring conscientiousness).

Key limitations of the current study include a lack of random assignment to LTM and MNP conditions (introducing a risk of selection bias) and a relatively modest number of LTMs. The available sample was below that for which the power analysis was conducted, which had assumed a moderate-to-large effect size (*d* = 0.74). Thus, it is very likely that the current study was underpowered to detect more modest effects. Our use of photographs may have obscured changes detectable through a thicker slice of behavior (e.g., video recordings) or behaviors with higher relevance to the constructs of interest. We also relied primarily on personality dimensions which may be less sensitive to short-term training, given their trait-like nature. While we focused on dimensions theoretically linked to meditation training and embodiment, we did not assess two of the five Big Five dimensions (i.e., extraversion, openness). Neither group of raters were experts in personality, so our results merely indicate a lay interpretation of the items that were rated, rather than a scientifically-based understanding of the specific personality dimensions assessed (although the Big Five items were drawn from self-report measures used to assess personality in the general population).[36] Further, the majority of our sample of raters were young, undergraduate students (mean age = 19.07). This group may be prone to a variety of perceiver effects that introduced bias into the ratings (e.g., associated with rating targets who are on average 30 years older), raising questions about the degree to which the ratings of photographs in the current study may generalize to interpersonal perceptions made by individuals of a wider variety of ages in the context of daily life.

These limitations notwithstanding, we believe this is the first study to demonstrate that naïve observers perceive LTMs differently than MNPs on several domains theoretically linked with mindfulness training, even through very minimal samples of behavior (i.e., still photographs). This work highlights the possibility that LTMs are perceived differently in daily life, which in theory could both represent an outcome of long-term training as well as a potential mechanism through which long-term training confers psychological and interpersonal benefits. Future work investigating the interpersonal effects of mindfulness training is warranted.

## Supporting information

S1 Table Sample R code for computing intraclass correlation coefficient (ICC3). (DOCX)
