# Supplementary material for: Still facial photographs of long-term meditators are perceived by naïve observers as less neurotic, more conscientious and more mindful than non-meditating controls
Source: PLoS One. 2019 Aug 28;14(8):e0221782. doi: 10.1371/journal.pone.0221782 (PMC6713443; doi:10.1371/journal.pone.0221782)
Supplement: S1 Table — (DOCX) [file pone.0221782.s001.docx]

**Supporting information**

**S1 Table. Sample R code for computing intraclass correlation coefficient (ICC3).**

vp <- function(data.g, formula.g) { # variance partitioning

lmer.out <- lmer(data = data.g, formula = formula.g)

var.residual<-attr(VarCorr(lmer.out), "sc")^2

var.comp <- ldply(VarCorr(lmer.out))

names(var.comp) <- c("Factor", "Variance")

var.comp <- rbind(var.comp, data.frame("Factor" = "Residual", "Variance" = var.residual))

var.comp$Percent <- round(var.comp$Variance / sum(var.comp$Variance) * 100, 1)

attr(var.comp, "mer") <- lmer.out

class(var.comp) <- c("data.frame", "G")

var.comp

}

#T1_calm

oneFac <- reshape(data=df[,c("SID",paste("WB",sids,"_T1_calm", sep=""))],

varying=paste("WB",sids,"_T1_calm", sep=""), v.names=c("rating"),timevar="Target",sep="_", direction="long")

oneFac$id <- NULL; oneFac$Rater <- factor(oneFac$SID); oneFac$Target <- factor(oneFac$Target)

lme1 <- lmer(data=oneFac, formula = rating ~ 1 + (1|Rater) + (1|Target))

vp1 <- vp(data=oneFac, formula=rating ~ 1 + (1|Rater) + (1|Target)); varT <- vp1$Variance[1]; varR <- vp1$Variance[2]; varRTe <- vp1$Variance[3]

varT/(varT + varRTe/table(table(df[!is.na(df$WB101_T1_calm),"SID"]))) #.84
